# Commission on Security and Cooperation in Europe (Helsinki Commission): Briefing on Truth, Reconciliation, and Healing Toward a Unified Future Thursday, July 18, 2019

**DOI:** 10.1089/heq.2021.29007.chr

**Published:** 2021-09-21

**Authors:** Gail C. Christopher

**Affiliations:** Executive Director, National Collaborative for Health Equity, Washington, District of Columbia, USA.

Chairman Hastings, Chairman Wicker, commission members, and our audience, thank you for holding this important briefing. I am honored to testify on methodologies that can unify and heal societies across the globe that have been divided by war, genocide, and other traumas reflecting a belief in a hierarchy of human value. My name is Gail C. Christopher. I am the founder of the Ntianu Center for Healing and Nature, the chairperson of the Trust for America's Health, and the architect and implementor of more than $1 billion in efforts spanning four decades to facilitate racial healing and jettison racism from American society.

Research reveals that the inequities caused by racism cost our nation almost $2 trillion annually in lost purchasing power, reduced job opportunities, and diminished productivity. Research also documents the extent that the conscious and unconscious belief in a racial hierarchy fuels the reluctance of political leaders and policymakers to acknowledge the inequities and devote adequate resources to addressing them. Our democracy, like others around the world, is based upon full human engagement and action on shared interests of the population. In order to move forward, this nation must heal the wounds of our past and learn to work together with civility, and indeed, with love. We must build the individual and collective capacity to “see ourselves in the face of the other.”

Our country has a history of enslaving people, committing genocide among Indigenous people, and embracing centuries of institutionalized racism. Yet, unlike other countries that have endured war, sectarian or racial strife, the United States has never undertaken a comprehensive Truth and Reconciliation Commission (TRC) effort to heal divisions and bring equal opportunities to all communities. Thus, America experiences a significant wealth gap between white families and families of color, the persistence of government-incentivized residential segregation, unequal access to quality health care and affordable housing, achievement gaps in education, and discrimination in hiring practices.

Throughout the world, extreme nationalism, racism, anti-Semitism, and other forms of ethnic and religious bias are often sustained by an antiquated notion that the human family can be divided and ranked based on physical characteristics and ascribed traits. These ill-conceived beliefs ossify, becoming hardened barriers among populations. This belief is alive today, as is the racism it has perpetuated and ingrained in America and other nations.

The planet has more refugees today than at any time on record, and the impacts of human conflict related to weakening multilateral institutions and rapid climate change will only increase the number. Across the globe, societies struggling with growing inequality and demographic changes are being offered scapegoats instead of solutions. It has proven far too easy for citizens to turn against families seeking a safer home, because anti-immigrant demagoguery taps into a well of beliefs that cast racialized Others and people in poverty as inferior and criminal. These are false beliefs. The truth is that, managed well, immigration makes societies stronger—and we never know when any of us will need welcome from a stranger.

When we uproot the false belief in a hierarchy of human value, we will be on firmer ground to face the challenges ahead. Together with other healing thought leaders, we have plotted a new course, one that can transform our nation as well as serve as a blueprint for other nations facing legacies of racism and discrimination. The Rx Racial Healing National Mobilization Campaign is a movement that aims to generate a critical mass of people committed to working together and healing the wounds of the past as we seek to end racism and the inequities it has created. Remember architect and systems thinker Buckminster Fuller once said:

“You never change things by fighting the existing model. You must create a new model that makes the existing model obsolete.”

By redefining racism as the embedded and entrenched belief system it is, Rx Racial Healing provides a needed on-ramp for launching a new model of relatedness that is grounded in the knowledge of our interconnected and equal worth as human beings. With this foundational idea in place, we can create new ways of living, policing, and governing, as well as ways of distributing resources more equitably because we see our collective common interests.

This campaign is empowering organizations reaching millions of people in every sector of nearly every community in our country to transform our society by going beyond just treating the symptoms of racism. Using a Rx Racial Healing methodology to create empathetic and compassionate support, our objective is to facilitate local action coalitions to jettison racial hierarchy and implement long-term policies and practices that address the impact of racial equity on health, education, housing, and economic opportunity.

The Rx Racial Healing vision identifies five imperatives for transforming communities:

- Leaders in the philanthropic, public, and private sectors should leverage media and technology to disseminate the new narrative about human origins and connectedness, informed by 21st century genomic science, to repudiate the false 17th century belief in separate and unequal human races. This public historical correction should include authentic narratives and experiences of diverse people who will provide previously untold historical and contemporary perspectives, fuel new understanding, and enhance capacity for self-compassion and empathy.
- We are already training a critical mass of facilitators in all disciplines, geographic areas, and organizations. They will provide Rx Racial Healing experiences for diverse groups to enhance skills and capacities for empathy, self-compassion, resilience, and perspective taking.

My final three recommendations are for policymakers.

- Congress and states should aim to overcome institutionalized racial separation patterns by implementing new approaches to land use, zoning, housing and transportation policies, mortgage finance, and resource development.
- Congress should review its public policies and administrative practices to ensure that they are honoring the humanity of all, and particularly redress past and current inequities in civil and criminal justice systems.
- Economic policymakers at every level of government should implement investment strategies that result in a more equitable economy that closes racial and ethnic income and wealth divides.

Rx Racial Healing is a 21st century approach to collective healing. It is the work of positively influencing the consciousness of a people to help create a world without the effects of racism and religious bias. While we are applying it to the inequities in the United States, it can be applied globally to address the various manifestations of the belief in human hierarchy in any society. *E pluribus unum*! Out of many, one. If America is to survive and to thrive as a democracy, we must begin to truly believe that we are one people, one human family. We must muster the courage to unlearn human hierarchy and act to redress the consequences of adhering to that false belief for centuries. We must learn to love one another, to show compassion and grace. The prescription for what ails us is racial healing.

## Rx Racial Healing Begins with a Change in Consciousness

By consciousness, I mean our beliefs and our states of awareness, both conscious and unconscious; particularly awareness and appreciation about the human family, our origin, and our sense of belonging and inter-relatedness. The idea of an interconnected human family is thwarted by the persistent belief in a hierarchical taxonomy of humanity; and the systemic vestiges of that antiquated belief that still mold our societal infrastructure and systems of democracy. Consciousness is not just thoughts. Consciousness encompasses emotions and feelings, as well as perceptions and attitudes that shape our beliefs and behaviors.

The Rx Racial Healing campaign is based on inter-related strategies: building a national organizational network and activating local action to promote racial healing and racial equity. At the national level, national partner organizations are using their leadership positions to engage others in their sectors to become champions for racial healing and equity. Organizations throughout the education, health, housing, economic development, philanthropic, faith, and nonprofit communities make up a second sphere of collaborating entities.

The goal is to help a critical mass of people work together to eradicate the false ideology of a hierarchy of human value and its harmful consequences. This is the change we are creating. We want to reach a critical mass, the minimal number of people needed to sustain a consciousness shift in our society away from permitted hatred, indifference, and lovelessness, toward unity and systemic human compassion for all. As such, the goal is education or re-education.

Rx Racial Healing enables people to conceptualize and experience a new model for relating as an extended human family, one that is capable of perspective taking and seeing ourselves in the face of the perceived “other,” feeling empathy, and demonstrating compassion with one another.

This outcome is achieved by engaging people in communities and organizations throughout the nation. Rx Racial Healing is a conceptual framework for action in communities and organizations which includes a specific racial healing circle methodology, which guides people from diverse backgrounds and perspectives through a story-telling process that leads them to recognize and embrace each other's humanity.

It is past time for calling out and eradicating the 17th century obsolete construct and belief in a hierarchy of human worth and value. It is now time to replace that old mental model with an accurate awareness and understanding of our common human ancestry and our equal interconnected humanity. This is the missing link needed for generating and sustaining an equitable social infrastructure in America and for realizing our aspirational vision for the promise of democracy.

When implemented on a large and comprehensive scale throughout the nation, Rx Racial Healing will help move us beyond needless divides toward the wholeness upon which a viable democracy depends. Why is this change so badly needed?

Our inability as individuals and as a society to value all human beings equally, or as Albert Einstein once said to “see ourselves in the face of the other,” is making us sick, literally.

Even more broadly speaking, the incapacity to value all human beings equally keeps us from experiencing optimal well-being and happiness. Our hearts and brains are designed to resonate with harmonious relationships. The opposite—fear and anxiety, separation, alienation, and hate—induces stress and distress. Distress causes a cascade of illness-related changes within our very cells in our physical bodies and within our body politic.

This inability is not unlike the design flaw in the Boeing 737 Max Jet airliner that is believed to have contributed to two plane crashes causing the needless tragic deaths of hundreds of passengers. Researchers estimate that 265 people die every day from racial health disparities in the United States. This is the equivalent of a 747 Jet crashing on a daily basis.

But it is not just people of color that suffer and die prematurely. The U.S. population, as a whole, lives shorter lives and has poorer health than our peer nations. Our residual belief in a false taxonomy and hierarchy of humanity—of human value or worth—is a major contributing factor to our poor health outcomes. Distress responses related directly and indirectly to racial fear, anxiety, and to its attendant social conditions contribute to hypertension and cardiovascular disease, glucose intolerance, and insulin resistance, and diabetes and its precursor metabolic syndrome. Dr. Jonathan Mezl's 2019 book *Dying of Whiteness* calculates the impact of public policies increasingly supported by white Americans and viewed through a white identity lens—such as the refusal to expand Medicaid in Tennessee or the loosening of gun laws in Missouri in the wake of Ferguson protests—on the population-level health of white Americans. Dr. Mezl finds that these two policies resulted in 10,506 lost years of productive white male life in Missouri and every single white resident in Tennessee 14.1 days of life.^1^

Researchers at Stanford University surveyed voters in the 2016 presidential election. Results showed that the majority of white Republican voters indicated fear of diversity was the primary reason for their vote. The undergirding belief system—racism—that devalues people based on perceived differences in physical characteristics like skin color, hair texture, and facial features is a foundational idea in America.

Our nation has so much to overcome.

The institution of slavery lasted throughout the formative centuries of the United States, 1619–1865 and officially ended because of the Civil War and the 13th Amendment to the U.S. constitution. However, former slave owners, state and local governments, and corporations created new ways to maintain the system of racial hierarchy. Journalist and author, Douglas Blackmon, wrote a Pulitzer Prize winning book on this in 2008, *Slavery by Another Name: The Re-Enslavement of African Americans from the Civil War to World War II*. Blackmon explores the brutal systems of convict leasing, share cropping, and peonage. These were all oppressive economic strategies to exploit and control emancipated African Americans.

The undergirding belief in a hierarchy of human value continued to define the culture of America well into the Twentieth Century. Other systemic manifestations included Jim Crow laws used to humiliate and deny social contact, residential and school segregation, overt discrimination across all public and private opportunity avenues; lynching and terror through racial violence perpetrated by hate filled individuals and organizations such as the Ku Klux Klan and White Citizen's councils. Beliefs die hard. Cultural norms die even harder, especially when they are embedded within all perceived authority, educational, protection, and survival mechanisms.

Such is the case for the fallacy of human hierarchy. This antiquated way of seeing and being with one another is a fossil formed during the 14th century and crystallized in the 18th century by Carl Linnaeus, Swedish Botanist, known as the father of taxonomy. But unlike other fossils, the belief in a hierarchy of human value still lives deeply in the hearts and mind of far too many people today. This idea must end and take its place in the museums like other historic relics.

The idea of a human value hierarchy must die now, before it kills us all!

Linnaeus first codified the scientific frame of human hierarchy and listed human “races” based on physical appearances and on continents of origin. He placed people like himself, Europeans, at the top of this hierarchy and other so called races in descending order of humanness—placing Africans at the very bottom of his hierarchical system.

Blackmon's book illustrates the lasting impact of the hierarchy created by Linneaus in the 1700s. Blackmon creates a new narrative by filling in decades of missing history about just how the belief in racial hierarchy was enforced well into the 20th century. It provides previously hidden information about an important albeit painful and tragic period in our nation's history. Yet, when he tells the stories in public forums, he leads with affirmation and context. He shows photos and reminds audiences that it was largely the unpaid labor of black men and women that cleared the dense forests to make way for railroads, highways, and metropolitan areas.

The protracted history of enslavement of African Americans and its aftermath often leads people to view racial hierarchy as only a black–white issue. To do so is a mistake. Linneaus' taxonomy reduced all people perceived as different from Europeans to the status of “less than” and the “other.”

Pigmentation or the lack there of are levers for social rejection or acceptance throughout the world. A dear colleague from India once told me that the first question families ask about the intended bride or groom is “How dark is the skin?” Skin lightening products are a multibillion-dollar global industry. Racial hierarchy beliefs have spawned colorism, prejudice, and discrimination against individuals with dark skin the world over. Often colorism manifests within racial and ethnic communities.

Twenty-first century science has ushered in a fresh awareness and understanding about human origins and genetic commonality. We all are 99.9 percent the same, having originally descended from a common human ancestry. This science should be the final nail in the coffin of belief in white superiority and its racialized hierarchy of human value. Instead, there is a resurgence in assertions of racist ideas under cover of legitimacy as nationalism and populism.

According the Children's Defense Fund's 2016 report, “The State of America's Children,” most of the children in our nation under age five are now children of color. In spite of this demographic reality, according to a 2019 Pew Research Center poll, 68 percent of Americans think race relations are getting worse in the United States. Hate crimes based on race, xenophobia, and religious intolerance are rising. Civility is declining at a time when our diversity is increasing.

The Rx Racial Healing Mobilization Campaign builds upon the Truth, Racial Healing, and Transformation (TRHT) process that I designed and launched with the W.K. Kellogg Foundation in 2017. The Rx Racial Healing campaign takes the next step—coupling the TRHT process and principles with a new over-reaching framework that enables the population to conceptualize and experience a new model for relating as an extended human family. The Rx Racial Healing framework is an adaptation of international TRCs that have been instrumental in resolving deeply rooted conflicts around the world, and underscoring the transformational power in healing the wounds of the past before progress can be made.

The TRC process is varied, but typically involves public and private activities designed to uncover and deepen the understanding of tragedies and/or human rights violations. Prior TRC efforts have been initiated by litigation, by government mandate, and by calls from activists. The TRC methodology is an international 20th century development involving public and private experiences for uncovering and deepening understanding of recent tragedies and human rights violations. The approach has been used previously to address historic wrongs in Australia, Canada, and a few communities in the United States.

## Personal Reflections on Racial Healing

I remember as a 15-year-old first beginning to understand the power of racism, and the need for healing.

Fate, luck, and talent took me from my all African American community in Cleveland and plopped me down into an all-white enclave, a summer arts encampment in Chautauqua, New York. Away from home for 6 weeks, I would have a roommate of a different race and be the only one-of-two people of color in the entire town.

Everyone seemed very nice and treated me well, but I did not even have a word for the sense of separation and alienation that I felt. I woke up very early every morning and walked, alone, to the wooded area in the small town. It was there that I discovered my love of nature and learned to appreciate the simple beauty of trees. I would sit on the picnic table listening to the sound of water flowing in a nearby brook, staring up at the oddly pale sky between the treetops for what seemed like hours.

Decades later, I would understand the science about the healing effects of nature, and how being within forested areas can actually help the body reduce levels of the stress hormone cortisol. I would become a champion for the global movement for engaging children with nature and open the Ntianu Center for Healing and Nature on a three-acre forested location in southern Maryland fed by an artesian spring.

Heavily scheduled days and evenings filled with concerts and shows made the weeks pass very quickly. Soon the once-in-a-lifetime summer arts experience drew to its end. On one of the camp's last days, as I walked past all the quaint Victorian houses on our little street, an ambulance appeared in front of our yellow house. Hurrying to see what was going on, I reached the front stairs in time to see my roommate being carried out on a stretcher. She was unconscious. I asked our house parents what had happened, and they told me that she had taken pills in an attempted suicide.

I ran up to our room, which suddenly seemed unbearably small. There I found a note she had written: “I don't want to go home. My father has taught me to hate black people. I now know that is a lie. I don't want to live like that anymore.” She had tried to take her own life.

The summer ended and I was never to learn her fate, but assumed they saved her life that day. I never forgot how it felt to have lived a brief moment within an innocent and authentic friendship which, unbeknownst to me, had pierced the veneer of racial hatred. Having come of age during the Civil Rights Movement era and having lived with both forced and de facto segregation, I, like so many of my peers, succeeded, in spite of the odds.

That summer long ago when, as a young girl, I came face to face with my roommate's deep pain, the child within me wanted to know why people believed in, taught, and acted upon hate. The adult and eventually the healer in me learned the answer to that question. I came to see, believe in, and know the power of love as a healing force.

I've spent the last 40 years translating that understanding into programs and social interventions to help make our lives, communities, and nation whole. I had experienced both the consequences of racial hatred and the courage to stand up for freedom that summer in Chautauqua.

## Racism Flows Like a River

Whether describing the Nile, Amazon, or Yangtze River, historians know that large rivers became the centers around which civilizations and nation states have flourished. This is true for the Mississippi river, named by the Indigenous dwellers of the Algonquin native tribes as the Father of Waters, the Mississippi River is one of the world's longest rivers. It touches 32 states in America. It is not an exaggeration to say that it is the river that became the center around which the United States flourished. Still today in the 21st century, the Mississippi river and its many tributaries drive up to 75% of the U.S. economy.

This mighty river provides a good metaphor for the power of a single phenomenon to shape our life and lives—the belief in a hierarchy of humanity value that flows through the American psyche and society like the Father of Waters, the Mississippi. It drove the slavery economy and became the center around which 18th, 19th, 20th, and even today's 21st century America flourishes.

Every river has a delta, a landform created from the earth and rocks along the banks that it touched while moving rapidly to the ocean beckoning its waters. The river carries this sediment and debris to an end place where movement slows to stagnation in the delta.

The human body has become the delta for the metaphorical river of racism. Sediment and debris from exposures have become socially embodied. Landforms—islands of separation, including residential segregation characterized by political and economic disinvestment—create adverse and toxic experiences for some, and fear of perceived “others.” These deltas help generate chronic stress and traumatic body responses which cause excess vulnerability to disease, and premature death.

But unlike rivers, whose existence and flow are vital for sustaining geographic and human life, racism is manmade. This antiquated belief system and way of seeing/being can be undone. Racism flows like a river, but it is not a river. Racism can and must be eliminated and its harmful consequences healed.

When implemented on a large and comprehensive scale throughout the nation, Rx Racial Healing will help move us beyond needless divides toward the wholeness upon which a viable democracy depends.
